# Pathogen Effector-Mediated Reprogramming of Plant Alternative Splicing: From Immune Regulation to Crop Disease Resistance

**DOI:** 10.3390/plants15162477

**Published:** 2026-08-15

**Authors:** Yunyun Li, Junru Mao, Song Kou

**Affiliations:** 1 National Engineering Research Center for the Development of Southwestern Endangered Medicinal Materials, Guangxi Botanical Garden of Medicinal Plants, Nanning 530023, China; 2 State Key Laboratory of Non-Food Biomass Energy Technology, Guangxi Academy of Sciences, Nanning 530007, China; lyy00795@hnu.edu.cn (Y.L.); maojunru0505@163.com (J.M.)

**Keywords:** pathogen effectors, alternative splicing, plant immunity, RNA processing, disease resistance

## Abstract

Plants have evolved sophisticated immune systems to defend against diverse pathogens, whereas pathogens deploy effectors to manipulate host cellular processes for successful infection. Recent studies have revealed that pathogen effectors can target host RNA processing, particularly pre-mRNA alternative splicing (AS), to reshape transcript isoform profiles and modulate plant immunity. However, the common principles and specific differences among pathogen effector-mediated regulation of host AS and their impacts on plant immunity have not been systematically summarized. In this review, we summarize recent advances in pathogen effector-mediated regulation of plant AS, focusing on three major mechanisms: targeting host splicing factors, altering RNA regulatory elements, and interfering with RNA-processing pathways. We discuss how effector-induced AS reprogramming affects immune-related gene expression and contributes to pathogen virulence. Furthermore, we highlight the emerging potential of AS regulation in disease resistance and disease-resistant crop breeding, and propose future directions for dissecting effector-specific splicing targets and translating AS-based regulatory mechanisms into crop improvement strategies. This review emphasizes host RNA splicing as an important regulatory layer in plant-pathogen interactions and provides new perspectives for understanding pathogen manipulation of plant immunity.

## 1. Introduction

Plants are continuously challenged by diverse pathogens, including fungi, oomycetes, bacteria, viruses, and nematodes [1,2,3]. To defend against pathogen invasion, plants rely on cell surface-localized pattern recognition receptors (PRRs) and intracellular nucleotide-binding leucine-rich repeat receptors (NLRs), which activate pattern-triggered immunity (PTI) and effector-triggered immunity (ETI), respectively [4,5,6]. Meanwhile, pathogens have evolved a variety of effector proteins that promote infection by interfering with host immune recognition, signal transduction, hormone homeostasis, and protein homeostasis [7,8,9].

In recent years, the host RNA processing machinery, particularly pre-mRNA alternative splicing (AS), has emerged as an important target manipulated by pathogen effectors to interfere with plant immunity [10,11,12,13,14,15]. AS enables a single gene to generate multiple mRNA isoforms, thereby regulating diverse aspects of gene function, including protein structure, subcellular localization, and transcript stability [16,17,18,19,20,21,22,23,24,25]. In plants, intron retention (IR) represents the most prevalent type of AS event and frequently introduces premature termination codons (PTCs), leading to transcript degradation through the nonsense-mediated mRNA decay (NMD) pathway or the production of truncated proteins [25,26].

Previous studies have shown that immune-related genes, including receptor-like kinases (RLKs/RLPs), NLRs, transcription factors, and hormone signaling components, frequently undergo AS changes during pathogen infection, indicating that AS is an important post-transcriptional mechanism for regulating plant defense responses [27,28,29,30,31,32,33,34]. For example, the tobacco receptor-like kinase Nt-Sd-RLK predominantly produces a truncated transcript lacking the kinase domain under unstimulated conditions, whereas lipopolysaccharide (LPS) treatment promotes the production of the full-length transcript, thereby activating downstream defense signaling [35].

**Figure 1:**
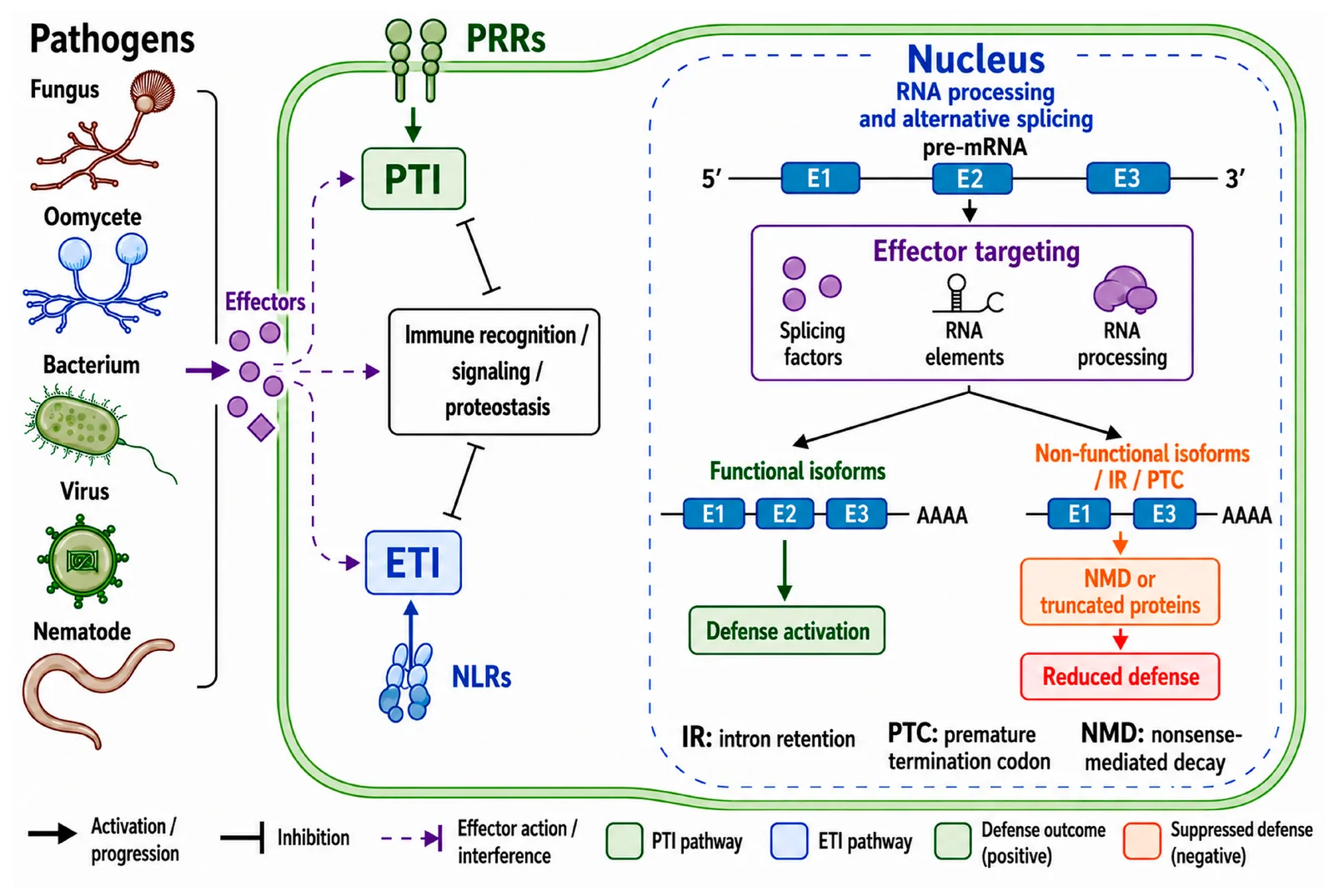
Overview of pathogen effector-mediated host alternative splicing reprogramming during plant immunity. Plants perceive pathogen invasion through pattern-recognition receptors (PRRs) and nucleotide-binding leucine-rich repeat receptors (NLRs), activating pattern-triggered immunity (PTI) and effector-triggered immunity (ETI), respectively. Pathogens interfere with host immune processes and RNA-processing pathways. By targeting splicing factors, RNA-regulatory elements, or RNA-processing pathways, pathogen effectors reshape host pre-mRNA AS, alter the balance of functional and non-functional isoforms of immune-related genes, and ultimately modulate plant defense outputs. E1–E3 indicate exon 1–exon 3, respectively. Solid arrows indicate activation or regulatory processes, whereas inhibitory lines indicate suppression.

These studies initially highlighted AS as an intrinsic regulatory mechanism by which plants actively modulate immune responses. However, with increasing understanding of pathogen effector functions, accumulating evidence has revealed that pathogens can actively interfere with or reprogram host pre-mRNA splicing processes through specific effectors [2,36]. Thus, AS is not only a post-transcriptional regulatory mechanism employed by plants to control immunity but also a potential target exploited by pathogens for host manipulation. The overall framework of pathogen effector-mediated host AS reprogramming and its impact on plant immunity is summarized in Figure 1.

For example, the *Phytophthora* effector PsAvr3c interacts with host SR-like splicing factors to alter the splicing patterns of defense-related genes [37], whereas the wheat stripe rust effector Pst-A23 directly binds RNA elements near pre-mRNA splice sites to influence splice-site selection [38]. Moreover, the *Ralstonia solanacearum* effector RipP2 targets the tomato splicing factor SR34a, further highlighting the ability of bacterial effectors to manipulate host splicing regulation [39]. Collectively, these studies suggest that diverse pathogens have evolved convergent strategies to reprogram the host splicing machinery.

Although several cases have revealed the phenomenon of effectors regulating host AS in recent years, this field is still developing. Many key questions remain unresolved, such as whether different pathogen effectors share common splicing regulatory patterns, how effectors select specific splicing targets, and how these AS changes further affect immune phenotypes. Furthermore, whether effector-sensitive AS events can serve as molecular markers for disease resistance or breeding targets warrants further exploration.

Based on this, this paper reviews recent research progress on pathogen effector-mediated plant AS reprogramming, focusing on summarizing the main mechanisms of action of effectors, host targets, and their impact on plant immunity. It also discusses the potential application value of effector-regulated AS in crop disease resistance improvement. By systematically reviewing this emerging research field, this paper aims to deepen our understanding of the mechanisms by which pathogens manipulate host post-transcriptional regulation and provide new insights for future disease resistance breeding strategies.

## 2. Basic Concepts of Alternative Splicing

Alternative splicing (AS) is a widely existing post-transcriptional regulatory mechanism in higher plants, which refers to the generation of multiple mRNA isoforms from the same pre-mRNA through different splicing methods, thereby expanding protein diversity and gene function regulation space [40,41,42,43,44].

**Figure 2:**
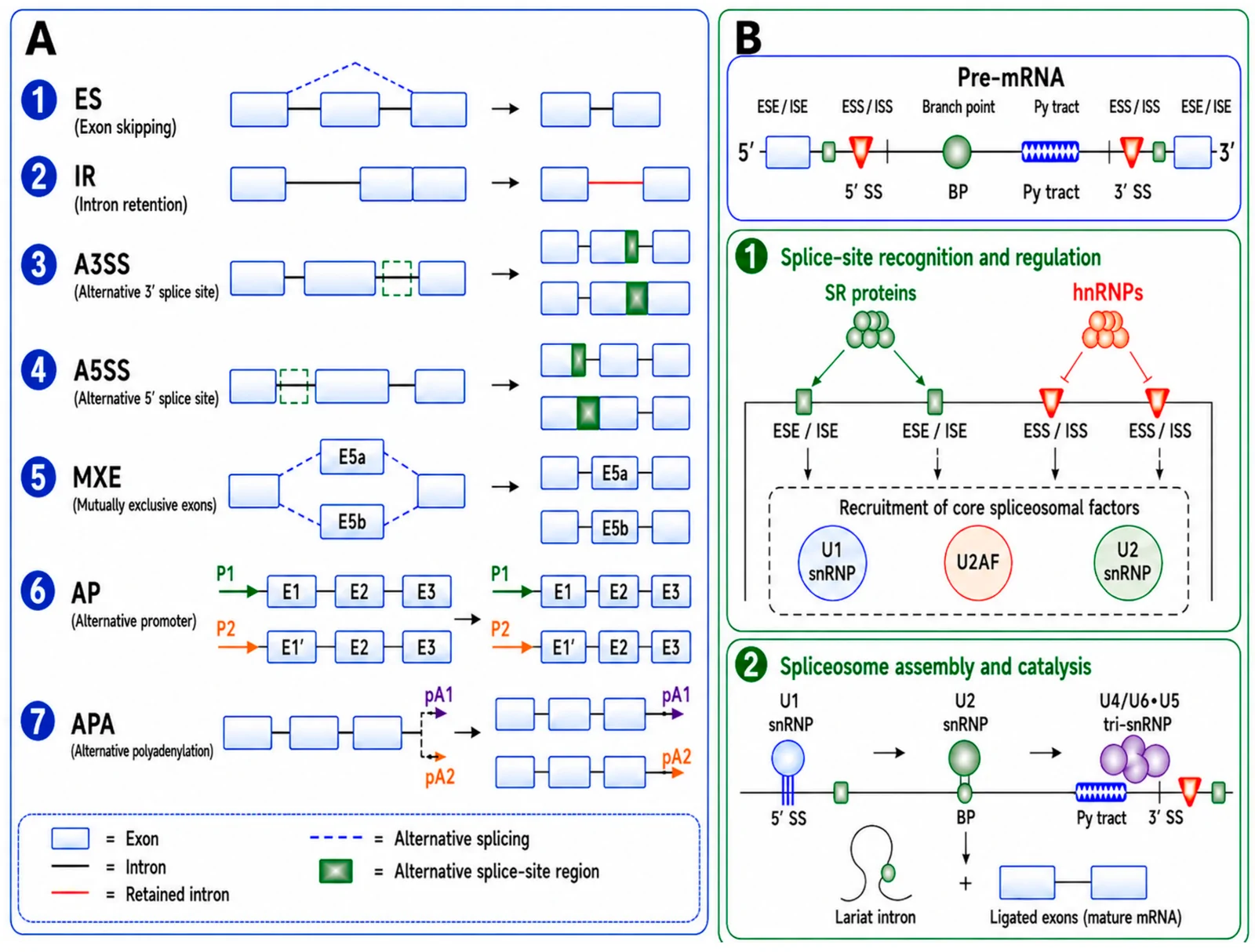
Plant alternative splicing types and spliceosome-mediated pre-mRNA processing. (**A**) Representative patterns of alternative transcript processing in plants, including exon skipping (ES), intron retention (IR), alternative 5′ splice-site selection (A5SS), alternative 3′ splice-site selection (A3SS), mutually exclusive exons (MXE), alternative promoter usage (AP), and alternative polyadenylation (APA). (**B**) Simplified model of splice-site recognition, regulation, and spliceosome assembly. U1 snRNP recognizes the 5′ splice site, U2 snRNP binds the branch point, and U2AF facilitates recognition of the polypyrimidine tract and 3′ splice-site region. Recruitment of the U4/U6·U5 tri-snRNP and subsequent spliceosomal rearrangements promote intron removal and exon ligation. SR proteins and hnRNPs interact with splicing enhancers or silencers to regulate spliceosome recruitment and transcript isoform production. SS, splice site; ESE/ISE, exonic/intronic splicing enhancer; ESS/ISS, exonic/intronic splicing silencer.

In plants, there are seven main types of AS (Figure 2A): exon skipping (ES), intron retention (IR), alternative 5′ splice site (A5SS), alternative 3′ splice site (A3SS), mutually exclusive exon (MXE), alternative promoter (AP), and alternative polyadenylation (APA) [10]. Intron retention (IR) is the most common in plants; for example, it accounts for over 47% of all AS events in rice genes [45] and approximately 56% in Arabidopsis genes [46]. Due to its high frequency and potential impact on transcript fate, IR represents an important regulatory mechanism contributing to plant transcriptome plasticity and immune adaptation.

Pre-mRNA splicing is catalyzed by the spliceosome (Figure 2B), a highly dynamic ribonucleoprotein complex composed of U1, U2, U4/U6, and U5 small nuclear ribonucleoproteins (snRNPs), together with numerous auxiliary splicing factors [47,48]. During the splicing process, U1 snRNP recognizes the 5′ splice site, whereas U2 snRNP binds to the branch point sequence and the surrounding region of the 3′ splice site. Subsequently, U4/U6 and U5 snRNPs are sequentially recruited and undergo extensive rearrangements to form a catalytically active spliceosomal complex, enabling intron removal and exon ligation [49,50]. The selection of splice sites is primarily controlled by splicing factors, among which serine/arginine-rich proteins (SR proteins) and heterogeneous nuclear ribonucleoproteins (hnRNPs) represent two major regulatory families. SR proteins generally promote splice-site recognition by binding to exonic or intronic splicing enhancers (ESEs/ISEs), whereas hnRNPs mainly bind to exonic or intronic splicing silencers (ESSs/ISSs) to repress exon inclusion [51,52,53]. Through competitive binding to cis-regulatory elements within pre-mRNAs, these splicing factors regulate the recruitment of core spliceosomal components, including U1 and U2 snRNPs [54], as well as auxiliary splicing factors such as U2AF (U2AF65/U2AF35), thereby regulating exon inclusion, intron retention, and splice-site selection [55].

The plant AS regulatory network dynamically adjusts the relative abundance of functional and non-functional transcript isoforms in response to diverse conditions, including developmental stages, environmental changes, and pathogen infection, thereby enabling precise regulation of gene expression and protein function. During plant immune responses, this highly dynamic post-transcriptional regulatory mechanism not only contributes to host defense regulation but also represents a potential target exploited by pathogen effectors for host manipulation and AS reprogramming.

## 3. Pathogen Effector-Mediated Alternative Splicing Regulation Mechanisms and Typical Examples

Recent studies have demonstrated that diverse pathogens have evolved specialized effectors that intervene in host RNA processing pathways. By reprogramming plant pre-mRNA AS, these effectors alter the isoform composition of immune-related genes, thereby modulating host defense outputs.

**Figure 3:**
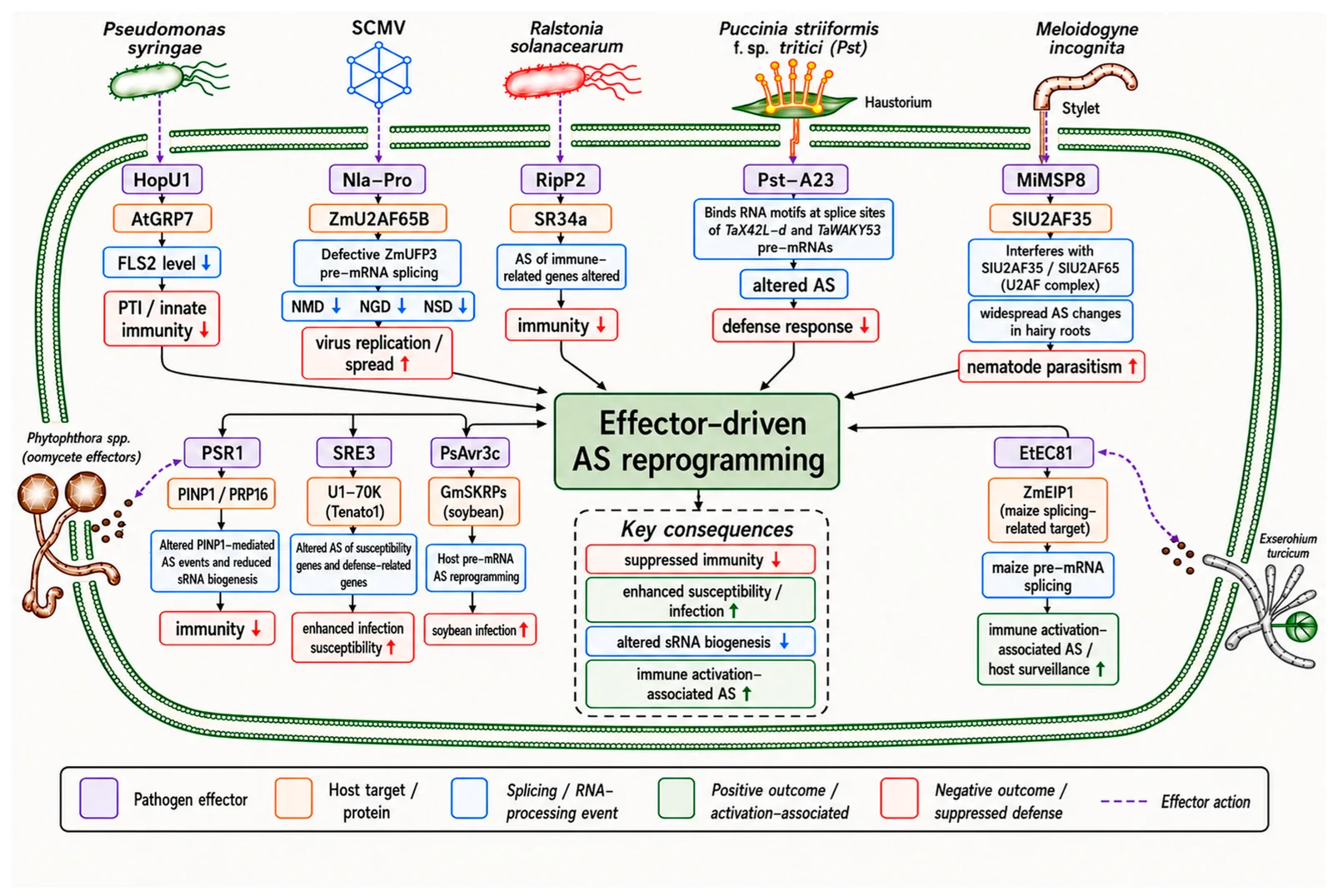
Pathogen effectors reprogram host pre-mRNA splicing to modulate plant immunity. Pathogens from different kingdoms deliver effectors that target host splicing factors, spliceosome-associated proteins, RNA elements, or RNA-processing pathways. Representative examples include HopU1, NIa-Pro, RipP2, Pst-A23, MiMSP8, the oomycete effectors PSR1, SRE3, and PsAvr3c, as well as EtEC81. These effectors alter immune-related alternative splicing (AS), RNA surveillance, or sRNA biogenesis, thereby suppressing immunity or enhancing susceptibility, whereas EtEC81 is associated with immune activation-related AS. Solid arrows indicate the direction or progression of regulatory events, and dashed purple connectors indicate effector action. Upward and downward arrows indicate increased and decreased levels or activities, respectively. SCMV, Sugarcane mosaic virus; NMD, nonsense-mediated mRNA decay; NGD, no-go decay; NSD, non-stop decay.

Based on currently available studies, we summarize reported cases of pathogen effector-mediated regulation of host AS into three representative strategies according to their molecular modes of action. First, effectors can target host splicing factors or spliceosome components, thereby interfering with splice-site recognition and spliceosome assembly. Second, effectors can directly bind RNA motifs located near splice sites of host pre-mRNAs, altering splice-site selection of specific transcripts. Third, effectors can indirectly influence the fate of immune-related transcripts by modulating RNA-binding proteins, small RNA biogenesis, or mRNA surveillance pathways. Collectively, these diverse mechanisms indicate that the host splicing machinery has emerged as a critical regulatory hub targeted by pathogen effectors during plant immune manipulation. Figure 3 summarizes the overall framework of pathogen effector-mediated reprogramming of host pre-mRNA AS.

Although different pathogen effectors exhibit distinct host targets and regulatory strategies, they commonly exploit key nodes within the host splicing regulatory network. Reported pathogen effectors involved in AS regulation, together with their host targets, regulatory mechanisms, and associated biological consequences, are summarized in Table 1.

### 3.1. Targeting Host Splicing Factors or Spliceosome Components

Effector-mediated targeting of host splicing factors represents one of the most extensively reported mechanisms of pathogen-induced AS regulation. Splicing factors play essential roles in splice-site recognition, spliceosome assembly, and alternative splice-site selection. Therefore, disruption of these factors by pathogen effectors has been shown to alter host pre-mRNA splicing patterns and affect the functional outputs of immune-related genes, as illustrated by the representative examples discussed below.

The *Phytophthora sojae* effector PsAvr3c represents one of the earliest reported examples of this mechanism. Previous studies demonstrated that PsAvr3c interacts with soybean splicing regulatory proteins GmSKRPs, enhances their accumulation in host cells, and alters the pre-mRNA splicing patterns of defense-related genes, thereby promoting *P. sojae* infection [37]. In this process, GmSKRPs function as host AS regulatory nodes involved in splice-site selection and transcript isoform regulation. This study provided the first direct evidence linking an oomycete effector to host splicing regulation, suggesting that pathogens can reshape plant immune outputs by interfering with host splicing regulators.

Similarly, the RXLR effector SRE3 from *Phytophthora infestans* targets the tomato splicing factor U1-70K. As an essential component of U1 small nuclear ribonucleoprotein (U1 snRNP), U1-70K participates in 5′ splice-site recognition during early spliceosome assembly. The interaction between SRE3 and U1-70K alters the AS patterns of tomato susceptibility- and resistance-related genes, thereby enhancing pathogen infection [56]. This finding indicates that pathogen effectors can target not only auxiliary splicing regulators but also core spliceosome-associated components to modulate immune-related transcript processing.

Bacterial effectors have also been shown to interfere with host splicing regulators. For example, the acetyltransferase effector RipP2 from *Ralstonia solanacearum* targets the tomato splicing factor SR34a and acetylates lysine 132 (K132) of SR34a, thereby altering the balance between functional and non-functional isoforms of immune positive regulators and attenuating host immunity. SR34a belongs to the serine/arginine-rich (SR) protein family, which generally functions in exon recognition and splice-site selection [39]. This example further demonstrates that pathogen-mediated manipulation of host splicing is not restricted to oomycete–plant interactions but also occurs in bacterial pathogenesis.

In addition, the root-knot nematode (*Meloidogyne incognita*) effector MiMSP8 interacts with tomato SlU2AF35 and interferes with the formation of the SlU2AF35–SlU2AF65 U2AF complex. The U2AF complex plays a critical role in 3′ splice-site recognition and represents an essential early step in spliceosome assembly. Disruption of U2AF complex formation by MiMSP8 results in widespread AS alterations in tomato hairy roots and facilitates nematode parasitism [57]. This study suggests that nematode effectors can also alter host transcript structures by disrupting spliceosome assembly, thereby creating favorable conditions for infection.

The *Exserohilum turcicum* effector EtEC81 represents another mode of effector-mediated targeting of spliceosome-associated regulatory proteins. EtEC81 interacts with the maize splicing-associated protein ZmEIP1 and remodels ZmEIP1-mediated pre-mRNA splicing regulation, leading to altered AS patterns and immune-associated splicing events in maize [58].

Collectively, pathogen effectors can target multiple layers of host splicing regulation, including SR proteins, U1-70K, U2AF complexes, and other spliceosome-associated regulators, to reprogram host pre-mRNA splicing. Although these targets differ among pathogens, they ultimately converge on a common outcome: altering the splicing patterns of immune-related genes and disrupting the balance between functional and non-functional transcript isoforms.

### 3.2. Direct Binding to RNA Motifs near Pre-mRNA Splice Sites

In addition to indirectly regulating host AS through targeting splicing factors, some pathogen effectors can directly interact with host RNA molecules. The stripe rust fungus (*Puccinia striiformis* f. sp. *tritici*) effector Pst-A23 represents a typical example of this mechanism. Studies have shown that Pst-A23 directly binds specific RNA motifs near the splice sites of wheat pre-mRNAs, including those of *TaXa21-H* and *TaWRKY53*, thereby altering their AS patterns and weakening host defense responses [38].

Traditionally, pathogen effectors have been considered to interfere with host immunity mainly through protein–protein interactions [62,63,64]. However, the discovery of Pst-A23-mediated RNA recognition demonstrates that effectors can directly recognize RNA elements and modulate splice-site selection independently of host splicing factors. This finding expands the functional repertoire of pathogen effectors and suggests that host pre-mRNAs themselves can serve as direct targets for pathogen manipulation. Therefore, the regulation of key immune gene splicing is not only controlled by endogenous splicing regulators but can also be directly hijacked by pathogen effectors.

### 3.3. Regulation of RNA-Binding Proteins, Small RNA Biogenesis, and mRNA Surveillance Systems

Some pathogen effectors do not directly target canonical splicing factors but instead indirectly alter host RNA fate and immune outputs by affecting RNA-binding proteins, small RNA (sRNA) biogenesis, or mRNA quality control pathways.

The *Pseudomonas syringae* effector HopU1 targets the Arabidopsis RNA-binding protein GRP7 and disrupts its interaction with the immune receptor FLS2 transcript, resulting in reduced FLS2 protein accumulation and suppression of pattern-triggered immunity (PTI) [59]. Although HopU1 is not a canonical example of effector-mediated AS reprogramming, this case highlights that pathogen effectors can also manipulate immune-related RNA fate through RNA-binding proteins, which represent important regulatory nodes in host RNA metabolism.

The *Phytophthora sojae* effector PSR1 provides a link between AS regulation and small RNA biogenesis. PSR1 targets the host splicing-associated RNA helicase PINP1/PRP16 and inhibits its RNA helicase and RNA-binding activities. Consequently, PSR1 disrupts sRNA biogenesis and RNA metabolism, leading to increased abnormal splicing events, including intron retention, and promoting pathogen infection [60].

During viral infection, the sugarcane mosaic virus (SCMV)-encoded effector NIa-Pro targets the maize splicing factor ZmU2AF65B. This interaction disrupts the association between ZmU2AF65B and the mRNA surveillance factor ZmUPF3 pre-mRNA, resulting in abnormal ZmUPF3 splicing and impairment of mRNA quality-control pathways, including nonsense-mediated mRNA decay (NMD), no-go decay (NGD), and nonstop decay (NSD). Consequently, RNA surveillance and antiviral immunity are compromised, facilitating viral infection [61].

### 3.4. Immune Consequences of Effector-Induced AS Alterations

The ultimate consequence of pathogen effector-mediated regulation of host AS is the alteration of immune-related transcript isoform composition, thereby influencing plant defense output [38,39,49,56,60].

In most cases, effector-induced AS reprogramming promotes pathogen infection. For example, effectors can reduce the abundance of full-length functional isoforms of immune positive regulators while increasing the production of intron-retaining or PTC-containing transcripts. These abnormal transcripts may be eliminated through nonsense-mediated NMD or translated into truncated proteins lacking essential functional domains [65,66]. Such changes ultimately compromise the activity of immune receptors, transcription factors, or signaling components, leading to weakened host defense.

However, effector-induced AS alterations do not always facilitate pathogen infection. Unlike most previously described effectors, the *Exserohilum turcicum* effector EtEC81 regulates maize pre-mRNA splicing and is associated with immune activation [58]. This finding suggests that effector-mediated perturbation of host splicing can also be perceived by plants and may function as a signal to trigger defense responses. Therefore, the host splicing machinery is not merely a target exploited by pathogens but may also serve as a potential surveillance system for detecting pathogen activities.

Overall, effector-induced AS alterations provide important insights into the molecular mechanisms underlying pathogen manipulation of host immunity. Moreover, these splicing events offer new opportunities to explore disease-resistance-associated regulatory features at the post-transcriptional level.

## 4. Implications of Effector-Mediated Regulation of Host Alternative Splicing for Disease-Resistance Breeding

**Figure 4:**
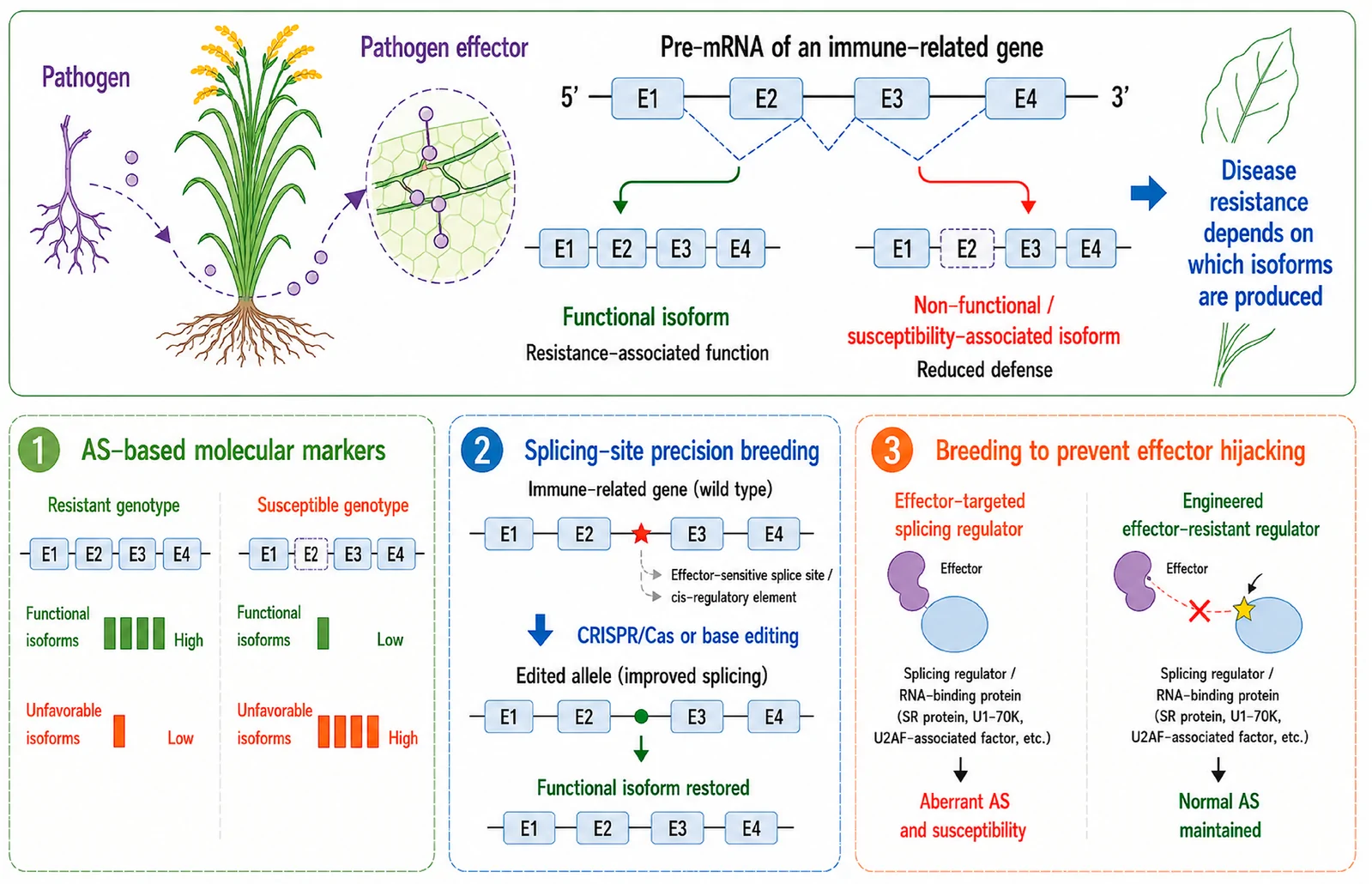
Effector-mediated alternative splicing reprogramming provides new strategies for disease-resistance breeding. Pathogen effectors reshape host AS patterns and alter the balance between functional and non-functional isoforms. (1) Effector-sensitive AS events and isoform ratios may serve as molecular markers for resistance evaluation. (2) Splice sites and cis-regulatory elements represent potential targets for precision breeding to restore favorable isoform production. (3) Engineering effector-resistant splicing regulators may reduce pathogen-mediated AS manipulation and enhance disease resistance.

Research on pathogen effector-mediated regulation of host AS has not only revealed a previously underappreciated pathogenic mechanism but also provided new perspectives for crop disease-resistance breeding [67,68]. Conventional breeding strategies mainly focus on the presence or absence of resistance genes, changes in gene expression levels, or sequence variations within protein-coding regions [69,70,71,72,73,74]. However, effector-mediated AS reprogramming suggests that the specific splice isoforms produced by immune-related genes, as well as the balance between functional and non-functional isoforms, may also play critical roles in determining plant disease resistance. Therefore, effector-sensitive AS events may represent important molecular features linking post-transcriptional regulation with disease-resistance phenotypes. These concepts provide new opportunities for disease-resistance breeding through multiple strategies (Figure 4).

First, effector-induced AS events may serve as potential molecular markers for evaluating disease resistance [75,76,77]. For certain immune-related genes, resistant genotypes may maintain a higher proportion of functional isoforms under pathogen infection or effector challenge, whereas susceptible genotypes may preferentially accumulate non-functional isoforms or susceptibility-associated transcripts. Therefore, isoform ratios may provide a more direct indication of gene functional status than conventional measurements of total transcript abundance [78,79]. By integrating transcriptome profiling with isoform-specific detection approaches, stable AS markers associated with resistance phenotypes could be identified and applied for germplasm evaluation and early screening of resistant materials.

Second, effector-sensitive splice sites and their cis-regulatory elements may represent promising targets for precision breeding [80,81,82,83]. When pathogen effectors alter splice-site selection, branch point recognition, or splicing regulatory elements to promote the production of unfavorable isoforms, genome-editing technologies such as CRISPR/Cas systems and base editing could be employed to precisely modify these regulatory regions [84]. Such strategies may increase the proportion of functional resistance-associated isoforms. Compared with directly disrupting gene function, splicing-based manipulation provides a more refined approach by optimizing immune outputs while preserving the essential biological functions of target genes [85]. This strategy may be particularly valuable for genes that simultaneously regulate plant growth and immunity.

In addition, host splicing factors and RNA-binding proteins targeted by pathogen effectors may represent promising candidates for breeding strategies aimed at preventing effector-mediated manipulation of host RNA processing. Previous studies have shown that splicing regulators, including SR proteins, U1-70K, and U2AF-associated factors, can be exploited by pathogen effectors to reprogram host alternative splicing (AS) [39,56,61]. By characterizing the interaction interfaces between effectors and host splicing regulators, key residues that reduce effector binding while maintaining normal splicing activity may be identified. Such modifications could generate crop varieties with reduced susceptibility to pathogen-mediated manipulation of the splicing machinery. However, because splicing factors generally regulate the processing of numerous transcripts, their genetic modification should be carefully evaluated through genome-wide AS profiling, developmental phenotyping, and disease-resistance assays to minimize unintended effects [86,87,88].

Collectively, pathogen effector-mediated regulation of host AS expands the focus of disease-resistance breeding from “whether a gene is expressed” to “which functional isoforms are produced from that gene.” This conceptual shift reveals regulatory layers that may be overlooked by conventional expression-based analyses and provides new molecular targets and strategies for crop disease-resistance improvement.

## 5. Conclusions

Alternative splicing represents an important post-transcriptional regulatory mechanism that links gene expression with functional diversification of immune-related proteins and plays a critical role in plant defense responses. This review highlights that pathogen effectors have evolved diverse strategies to manipulate host alternative splicing by targeting splicing factors, recognizing RNA regulatory elements, or interfering with RNA-processing pathways. Through these mechanisms, pathogen effectors can reshape the splicing patterns of immune-related genes, alter transcript isoform composition, and ultimately modulate plant immune outputs. These findings establish host splicing machinery as an important regulatory layer exploited by pathogens during plant–pathogen interactions. Moreover, effector-mediated AS reprogramming provides new insights into the post-transcriptional regulation of disease resistance and highlights the potential of manipulating key splicing regulators or immune-associated isoforms for future crop improvement.

## 6. Perspectives

Although increasing evidence indicates that pathogen effectors can manipulate host alternative splicing, our understanding of effector-mediated AS reprogramming remains at an early stage. Current studies have mainly focused on identifying effector targets and differential splicing events, whereas several fundamental questions remain unresolved. It is still unclear how specific AS alterations contribute to immune phenotypes, how effectors selectively recognize and regulate particular splicing targets, and how actively driven effector-mediated AS reprogramming can be distinguished from secondary splicing changes associated with pathogen infection. Future studies integrating long-read transcriptome sequencing, RNA interaction analyses, and functional characterization of individual isoforms will be essential for establishing causal relationships among effector targets, splicing alterations, isoform functions, and immune outputs.

Moreover, effector-mediated AS reprogramming provides a new conceptual framework for crop disease-resistance improvement. Unlike conventional breeding strategies that mainly focus on resistance genes, gene expression changes, or coding sequence variation, studies of AS highlight the critical contribution of post-transcriptional regulation to plant immune outputs and disease-resistance phenotypes. Manipulating the production and relative abundance of functional splice isoforms may therefore provide new opportunities to enhance crop disease resistance. Integrating effector-responsive splicing regulatory networks with genome editing, multi-omics analyses, and molecular breeding strategies may facilitate the development of disease-resistant crops based on post-transcriptional regulatory mechanisms.

## Figures and Tables

**Table 1:** Pathogen effectors that regulate host alternative splicing and their biological functions.

Pathogen Type	Effector	Host Target	Mechanism/Evidence	Immune Outcome	References
Oomycete	PsAvr3c	GmSKRP1/2	Target splice-related protein, reprogram AS	Promote infection	[37]
Fungus (Basidiomycota)	Pst_A23	pre-mRNA RNA motif	Direct binding to splice site RNA motif (RNA-AS)	Suppress immunity	[38]
Bacteria	RipP2	SR34a	Target splice factor, reprogram AS	Promote infection	[39]
Oomycete	SRE3	U1-70K	Target core spliceosome component, reprogram AS	Enhance infection	[56]
Nematode	MIMSP8	SIU2AF35	U2AF complex disruption	promote parasitism	[57]
Fungus (Ascomycota)	EtEC81	ZmEIP1	Target splice-related protein, reprogram AS	Activate immunity	[58]
Bacterium	HopU1	GRP7	Target RNA-binding protein	Suppress PTI	[59]
Oomycete	PSR1	PINP1	Interfere splice factor + sRNA (AS/sRNA)	Suppress immunity	[60]
Virus	NIa-Pro	ZmU2AF65B	Inhibit splice factor, disrupt mRNA surveillance	Promote viral replication	[61]

Note: AS, alternative splicing; SR proteins, serine/arginine-rich proteins; U2AF, U2 auxiliary factor.

## Data Availability

The data of this study are available from the correspondence author upon reasonable request.
